# Bottlenose Dolphin (*Tursiops truncatus)* Whistle Modulation during a Trawl Bycatch Event in the Adriatic Sea

**DOI:** 10.3390/ani11123593

**Published:** 2021-12-19

**Authors:** Valentina Corrias, Giovanni de Vincenzi, Maria Ceraulo, Virginia Sciacca, Antonello Sala, Giuseppe Andrea de Lucia, Francesco Filiciotto

**Affiliations:** 1 Institute of Polar Sciences-National Research Council (CNR-ISP), 98122 Messina, Italy; virg.sciacca@gmail.com (V.S.); francesco.filiciotto@cnr.it (F.F.); 2 Department of Ecology and Biology (DEB), University of Tuscia, 01100 Viterbo, Italy; 3 Institute of Anthropic Impact and Sustainability in Marine Environment-National Research Council (CNR-IAS), 09170 Oristano, Italy; giuseppe.delucia@cnr.it; 4 eConscience—Art of Soundscape, Via Provinciale 610, 90046 Palermo, Italy; gioggio.devincenzi@gmail.com; 5 Institute of Anthropic Impact and Sustainability in Marine Environment-National Research Council (CNR-IAS), 91021 Trapani, Italy; ceraulo.maria@gmail.com; 6 Institute for Marine Biological Resources and Biotechnologies-National Research Council (CNR-IRBIM), 60125 Ancona, Italy; antonello.sala@cnr.it

**Keywords:** bottlenose dolphin, whistle, bycatch, signature, acoustic communication, stress

## Abstract

**Simple Summary:**

There is some evidence that the presence of dolphins in fishing areas represents a concrete economic loss for fishermen due to their depredation activities on the entangled fish on the nets. Bycatch events are one of the major sources of anthropogenic mortality of species of conservation interest in the world. *T. truncatus* is a plastic species and the more frequently observed species in the Adriatic Sea owing to the natural tendency to interact with the fishing activities in the area. This case report describes the acoustic parameters detected in whistle spectral contours associated with low-frequency signals recorded with a passive acoustic monitoring device in an exceptional event of bycatch that involved three individuals during a midwater commercial trawling in the Adriatic Sea.

**Abstract:**

Marine mammal vocal elements have been investigated for decades to assess whether they correlate with stress levels or stress indicators. Due to their acoustic plasticity, the interpretation of dolphins’ acoustic signals of has been studied most extensively. This work describes the acoustic parameters detected in whistle spectral contours, collected using passive acoustic monitoring (PAM), in a bycatch event that involved three Bottlenose dolphins during midwater commercial trawling. The results indicate a total number of 23 upsweep whistles recorded during the bycatch event, that were analyzed based on the acoustic parameters as follows: (Median; 25th percentile; 75th percentile) D_r_ (second), total duration (1.09; 0.88; 1.24); f_min_ (HZ), minimum frequency (5836.4; 5635.3; 5967.1); f_max_ (HZ), maximum frequency, (11,610 ± 11,293; 11,810); f_c_ (HZ), central frequency; (8665.2; 8492.9; 8982.8); BW (HZ), bandwidth (5836.4; 5635.3; 5967.1); Step, number of step (5; 4; 6). Furthermore, our data show that vocal production during the capture event was characterized by an undescribed to date combination of two signals, an ascending whistle (upsweep), and a pulsed signal that we called “low-frequency signal” in the frequency band between 4.5 and 7 kHz. This capture event reveals a novel aspect of *T. truncatus* acoustic communication, it confirms their acoustic plasticity, and suggests that states of discomfort are conveyed through their acoustic repertoire.

## 1. Introduction

Marine mammals have evolved the most sophisticated and specialized structures for producing and receiving sounds. Dolphins (family: Delphinidae) use acoustic signals to coordinate movements and social behavior [1] as well as in cooperative effort, navigation, and foraging [2,3]. The acoustic plasticity of dolphins [4] is revealed by their ability to imitate the vocalizations of conspecifics [5], to modify signals in relation to environmental and anthropogenic noise [4,6], and to emit different signals in relation to different behaviors [7]. Delphinids, the family where the interpretation of acoustic signals has been studied most extensively, produce a variety of sounds that can be classified into two main categories: rapid repetition rate echolocation clicks, click trains, burst, and tonal frequency modulated whistles [8,9,10]. According to some studies, whistles and burst pulses are predominant in social contexts [9,11]. Alterations in whistle parameters may indicate high variation in the message conveyed and reflect changes in the transmission of emotional information [12]. With regard to the latter feature, changes in marine mammal vocal structures have been investigated to assess whether they correlate with stress levels [13]. In a study on the ability of some whistle parameters to serve as stress indicators in *T. truncatus*, Esch et al. [14] reported a higher whistle rate and number of loops during brief capture–release events compared to undisturbed conditions. *T. truncatus* is probably the most common cetacean species in the Adriatic Sea [15,16], and is protected by species and habitat conservation laws and international agreements. Its interaction with fisheries mostly consisting of opportunistic foraging have been documented by several studies [17,18,19]. According to several reports, *T. truncatus* specimens can learn to take fish from trawls, gillnets, and aquaculture cages, suggesting that this behavior can generate a dependence on human activities. Bycatch can occur as a consequence of these strong interactions and may affect the survival of this vulnerable species [20] in the Mediterranean Sea. The northern Adriatic Sea is characterized by a dense presence of commercial pelagic trawlers, whose nets are usually towed at a relatively high speed, with unpredictable route changes that enhance the scope for entanglement [21]. The main target species of midwater trawlers are *Engraulis encrasicolus* and *Sardina pilchardus,* which are among the major components of the bottlenose dolphin diet. In this area, *T. truncatus* is the most frequently observed marine mammal species and the only one with a propensity to interact with human activities. In this case report, we describe the acoustic parameters detected in whistle spectral contours associated with low frequency signals, during an exceptional bycatch event that involved three bottlenose individuals during a midwater commercial trawling with fatal consequences for the three specimens.

## 2. Materials and Methods

Since 2006, an extensive monitoring program of bycatch of long-lived species like cetaceans, sea turtles, and elasmobranchs by Italian midwater pair trawlers has been conducted in the northern central Adriatic Sea [22,23]. The information collected in its framework provides a unique opportunity to assess the operational details of capture events and the abundance trends of species over time [24].

Eleven acoustic monitoring surveys were carried out from March to June 2017 during commercial fishing activities onboard two midwater pair trawlers activities.

Twenty-eight hours of acoustic data recordings were obtained in the course of 51 trawling operations. Surveys were conducted only in good sea conditions. Bottlenose dolphins’ acoustics vocalizations were collected and behavioral events were observed throughout all the fishing operations by using the focal group sampling [25]. The acoustic data were collected using an autonomous recorder secured to the headrope and oriented towards the trawl codend, opposite to the direction of towing. The weights ensured that the net was towed at an average depth of 20 m. The equipment included a calibrated omnidirectional hydrophone with a flat sensitivity response of −174.5 (±2) dB re V/μ Pa from 0.1 to 100 kHz (Low Noise Broadband Hydrophone BII 7016 T6, Benthowave Instrument Inc., Collingwood, ON, Canada) and a digital signal processor (mod. C5535 DSP-TMS320C5535) coupled to an AIC3204 audio codec (both from Texas Instruments, Dallas, TX, USA). The sampling rate was 192 kHz, resolution was 16 bits. Recording sessions lasted about 60 min.

**Figure 1:**
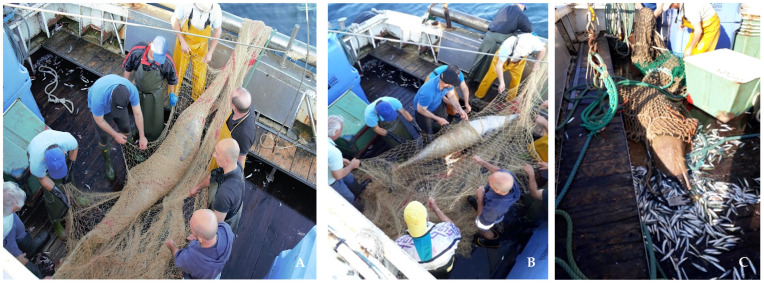
The three bycatches during the disentanglement operations, two were wrapped in the fishing net (**A**,**B**) and one inside the codend (**C**). Gulf of Venice, 18 May 2017 h 7:00 a.m., 45°18826′ N–12°41804′.

All acoustic data thus recorded (28 h) were visualized (*n* = 841) and the whistle parameters analyzed using RX5 audio editor software (iZotope, Cambridge, MA, USA) with a Fast Fourier Transform (FFT) size of 2048 points, an overlap of 50% and Hann window. The instantaneous sampling protocol of focal group behavior (natural, opportunistic feeding, socializations, and travel) [25] was used in each observation event involving interactions between bottlenose dolphins and fishing operations. Information such as group size and life stage of individuals (calf, sub-adult, adult) was also recorded. During sightings, dorsal fin features were collected using the photo-identification method [26]. This report describes a fishing trip where a group of three dolphins (2 adults and 1 sub-adult) was captured when interacting with a commercial midwater trawl pair. The parallel movement of the two ships in the hauling phase prevented the escape of the dolphins so that the three individuals were caught in the net and died (Figure 1). Anatomical observations indicated that all the individuals were females. None of them showed physical signs of external trauma following the direct action of the nets, the only visible sign was the foam present on the blowhole as a consequence of drowning asphyxiation. Visual inspection of the spectrograms allowed to identify several types of whistle contours; whistles recorded during the hauling of the bycatch event (*n* = 23) were all of the ascending type (upsweep), i.e., signals whose spectral contour is mostly ascending without negative inflection points in frequency modulation. The acoustic parameters measured in the spectrogram of each upsweep signal included duration (D_r_, s), total duration of the vocalization; minimum frequency (f_min_, Hz), minimum frequency value of the vocalization out of the total vocalization; maximum frequency (f_max_, Hz): maximum frequency value of the vocalization in the total vocalization; bandwidth (BW, Hz): f_max_–f_min_ range; central frequency (f_c_, Hz); frequency value in the middle portion of the bandwidth; and number of steps, according to recent studies about the acoustic emissions of marine mammals [14,27]. The STATISTICA 7.1 (StatSoft, TIBCO Software Inc., Palo Alto, CA, USA) software package was used for statistical analysis.

## 3. Results

**Figure 2:**
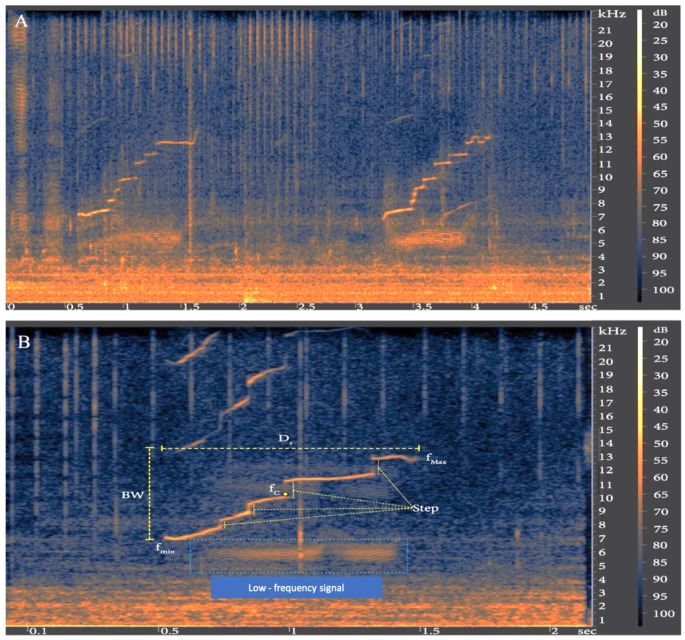
Spectrograms (FFT size 2048-point, Hann window, linear frequency scale) of upsweep whistles of *T. truncatus* recorded during the bycatch event. (**A**) A sequence of two upsweeps. (**B**) Detail of an upsweep showing the parameters analyzed: D_r_, total duration; f_min_, minimum frequency; f_max_, maximum frequency; f_c_, central frequency; BW, bandwidth; Step. The low-frequency bursts are highlighted within the dotted blue rectangle.

A total of 23 upsweep whistles were recorded during the bycatch event (Figure 2A). The whistle contour showed a clear step-like profile where vertical narrow frequencies were preceded and followed by a flat upsweep (Figure 2B). The median, 25th and 75th percentiles of all whistle parameters are reported in Table 1. These upsweep whistles displayed a simultaneous distinctive impulse signal, that we denominated “low-frequency signal”, which consisted of low-frequency bursts. Their frequency band ranged from 4.5 to 7 kHz (Figure 2A,B). These kinds of more complex associated signals occurred exclusively during the bycatch event.

From the matching of the dorsal fins, no one of the three individuals were sighted before the bycatch event.

## 4. Discussion

The ability to transmit information to conspecifics is crucial for dolphins, which live in communities with highly complex social structures [8]. Acoustic signals play an important role in both species and group recognition, although the degree of signal modulation varies widely among populations [28]. Notably, variations in whistle characteristics may reflect inter-individual variation or changes in the transmission of emotional information [29]. According to [12], whistle acoustic parameters can vary independently under a variety of stimuli, including stressful events. This study describes the whistle structure of the bottlenose dolphin in relation to a bycatch event pointing out that acoustic parameters and contours may vary in response to the context and be associated with other signals. These findings are in line with early works on the function of dolphin whistles that attempted to associate discrete whistle contours with behavioral state such as fright or disturbance [14,30]. In a study assessing whether the bottlenose dolphin signature whistles served as indicators of stress or stress levels, Esch et al. [13] reported that whistle duration changed both due to stressful events and in relation to group structure and composition (age, sex, mother–calf communication). Our findings suggest that the change in whistle acoustic parameters, recorded during a bycatch event, conveyed to conspecifics also the distress due to the entrapment in the net. Our data show that vocal production during the catch event was characterized by a combination of two signals, an ascending whistle (upsweep) and low-frequency signal (burst). Their association has never been described in *T. truncatus,* despite its large, complex, and extensively investigated repertoire of whistles and pulsed sounds. As burst pulsed sounds have been associated with aggressive [31,32,33] or socializing behaviors [1,34], the combination of bursts and whistles that we found in our study could suggests a different meaning to the intra-specific communication message, probably related to a distress event. This capture event reveals a further aspect of *T. truncatus* acoustic communication and it confirms the complexity of its repertoire suggesting that states of discomfort are conveyed through their acoustic emissions.

## 5. Conclusions

This case report describes for the first time a novel aspect of the acoustic communication of *T. truncatus* that occurred during a trawl bycatch event in the Adriatic Sea. We assume that dolphins modulate the contours and harmonics composition of the whistles and that pulse signals bursts play a social role that has not yet been fully explored. Further acoustic data of dolphin interactions with fishing nets are needed to determine how the emission patterns of the low-frequency signal can be associated with whistles in aggressive or distress contexts. The use of passive acoustics monitoring (PAM) allowed us to obtain unique information on the acoustic repertoire of *T. truncatus* in a highly man-made coastal environment. This work aims to contribute to the definition of a protocol in collaboration with the authorities, in order to implement monitoring that provides for the early detection of possible state of stress and to prevent bycatch events.

## Figures and Tables

**Table 1:** Whistle parameters (median; 25th and 75th percentile), assessed during the catch event (*n* = 23): D_r_, total duration (s); f_max_, maximum frequency (HZ); f_min_, minimum frequency (HZ); f_c_, central frequency (HZ); BW, bandwidth (HZ); Step, number of steps.

Parameters	Units	Catch Event (*n* = 23)	
		Median	25–75 Percentile
D_r_	(second)	1.09	0.88–1.24
f_min_	(Hertz)	5836.4	5635.3–5976.1
f_max_	(Hertz)	11,610	11,293–11,810
f_c_	(Hertz)	8665.2	8492.9–8982.8
BW	(Hertz)	5944.1	5600.2–6174.3
Step	Number	5	4–6

## Data Availability

Data is contained within the article.
